# Long-term omega-3 supplementation modulates behavior, hippocampal fatty acid concentration, neuronal progenitor proliferation and central TNF-α expression in 7 month old unchallenged mice

**DOI:** 10.3389/fncel.2014.00399

**Published:** 2014-11-21

**Authors:** Trent Grundy, Catherine Toben, Emily J. Jaehne, Frances Corrigan, Bernhard T. Baune

**Affiliations:** ^1^Discipline of Psychiatry, School of Medicine, University of AdelaideAdelaide, SA, Australia; ^2^School of Medicine and Dentistry, James Cook UniversityTownsville, QLD, Australia

**Keywords:** polyunsaturated fatty acid, omega 3, TNF-α, neurogenesis, cognition

## Abstract

Dietary polyunsaturated fatty acid (PUFA) manipulation is being investigated as a potential therapeutic supplement to reduce the risk of developing age-related cognitive decline (ARCD). Animal studies suggest that high omega (Ω)-3 and low Ω-6 dietary content reduces cognitive decline by decreasing central nervous system (CNS) inflammation and modifying neuroimmune activity. However, no previous studies have investigated the long term effects of Ω-3 and Ω-6 dietary levels in healthy aging mice leaving the important question about the preventive effects of Ω-3 and Ω-6 on behavior and underlying molecular pathways unaddressed. We aimed to investigate the efficacy of long-term Ω-3 and Ω-6 PUFA dietary supplementation in mature adult C57BL/6 mice. We measured the effect of low, medium, and high Ω-3:Ω-6 dietary ratio, given from the age of 3–7 months, on anxiety and cognition-like behavior, hippocampal tissue expression of TNF-α, markers of neuronal progenitor proliferation and gliogenesis and serum cytokine concentration. Our results show that a higher Ω-3:Ω-6 PUFA diet ratio increased hippocampal PUFA, increased anxiety, improved hippocampal dependent spatial memory and reduced hippocampal TNF-α levels compared to a low Ω-3:Ω-6 diet. Furthermore, serum TNF-α concentration was reduced in the higher Ω-3:Ω-6 PUFA ratio supplementation group while expression of the neuronal progenitor proliferation markers KI67 and doublecortin (DCX) was increased in the dentate gyrus as opposed to the low Ω-3:Ω-6 group. Conversely, Ω-3:Ω-6 dietary PUFA ratio had no significant effect on astrocyte or microglia number or cell death in the dentate gyrus. These results suggest that supplementation of PUFAs may delay aging effects on cognitive function in unchallenged mature adult C57BL/6 mice. This effect is possibly induced by increasing neuronal progenitor proliferation and reducing TNF-α.

## Introduction

The role of diet in the maintenance of mental health has long been recognized. Evidence suggests that the typical western diet is detrimental to the maintenance of cognitive function with aging (Francis and Stevenson, 2013). Recently, dietary interventions aimed at preventing cognitive decline have received attention, as it appears that diet across the entire lifespan plays a complex role in the development of cognitive decline (Gillette-Guyonnet et al., 2013). This suggests that early intervention, for example, up to middle age, may have more substantial benefits on cognitive function in later life as opposed to interventions in the elderly only.

Clinical trials have established that supplementation with omega (Ω)-3, or a combination of Ω-3 and Ω-6 may have a role in the management and prevention of mild cognitive impairment (MCI) (Yehuda et al., 1996; Freund-Levi et al., 2006; Chiu et al., 2008; Yurko-Mauro et al., 2010). Animal studies demonstrate improved spatial recognition memory in aged mice after 7 weeks or 2 months of Ω-3 polyunsaturated fatty acid (PUFA) supplementation (Jiang et al., 2009; Labrousse et al., 2012). Previous investigation into the mechanisms of this effect suggest that Ω-3 PUFA supplementation has anti-inflammatory properties in aged mice (Labrousse et al., 2012) and increases neurogenesis in young mice (Kawakita et al., 2006), through stimulating brain derived neurotrophic factor (BDNF) and tropomyosin receptor kinase B (TrkB) expression (Jiang et al., 2009; Valente et al., 2009; Bhatia et al., 2011). Furthermore, it appears to reduce neuronal apoptosis after induced hypoxic injury (Zhang et al., 2010) through increasing anti-apoptotic proteins (Bcl-xl, Bcl-2, Bfl-1), while decreasing pro-apoptotic proteins (Bax, Bik) (Lukiw et al., 2005). It is also important to note that increased Ω-6, or a low ratio of Ω3:Ω6, is also associated with impaired cognition and behavior in animal and human studies (Loef and Walach, 2013; Van Elst et al., 2014), although few studies have looked at this to date.

Thus, far the effects of dietary intervention on cognition in mature mice up to 7 months of age, before the onset of any measurable impairment, have yet to be investigated. Furthermore, the majority of studies investigating Ω-3 and Ω-6 PUFA supplementation in animal models of cognitive dysfunction have used short term (≤2 months) supplementation (Minogue et al., 2007; Petursdottir et al., 2008; Suchy et al., 2009; Labrousse et al., 2012), or Ω-3 supplementation in animals previously deficient in Ω-3 (Hashimoto et al., 2002, 2005, 2009; McNamara et al., 2010). A recent systematic review suggested that long term Ω-3 supplementation (>10% of animals lifespan) was most effective in reducing amyloid pathology and improving cognitive function (Hooijmans et al., 2012). Currently, studies that have examined a longer period of supplementation (3–12 months) have largely been limited to murine models of genetic predisposition to AD (Calon et al., 2004, 2005; Oksman et al., 2006; Arendash et al., 2007; Hooijmans et al., 2007; Arsenault et al., 2011). One such study in APPswe/PS1ΔE9 mice over 7 months found no significant protective effect (Arendash et al., 2007), while another study in 3xTG-AD mice identified that a high Ω-3 diet improved exploration time (Arsenault et al., 2011). As diet is likely to affect predisposition to cognitive decline across the lifespan, the effects of modifying Ω-3-Ω-6 dietary intake supplementation until close to middle age (7 months of age) is important to consider.

Our study aimed to examine the preventative effect of long-term (~60% of lifetime) high Ω-3:Ω-6 dietary supplementation on predisposition to cognitive decline, and to examine the underlying mechanisms of this effect. While many people may only start to increase their intake of Ω-3 dietary supplementation later in life, longer periods of supplementation may be more beneficial in the prevention of chronic disease such as cognitive decline (Simopoulos, 2011). This study utilized three different diets, a low Ω-3:Ω-6 ratio diet, a medium Ω-3:Ω-6 ratio diet and a high Ω-3:Ω-6 ratio diet. Thus, the dose-related and Ω-3:Ω-6 ratio related effects of PUFA dietary modification on cognition-like behavior, neuroinflammation and neuronal progenitor proliferation in C57BL/6 mice were investigated.

## Methods and materials

### Animals and diet

Male (*n* = 18) and female (*n* = 12) C57BL/6NHsd mice, starting age 3 months, were used throughout the experiments. *Ad libitum* fed mice were housed in Laboratory Animal Services (LAS) Adelaide approved conditions with 3–5 animals per cage in individually ventilated cages (IVC) with a 12 h light/dark cycle. Handling and a general health examination including weighing of mice occurred twice per week. This study received ethics approval from the University of Adelaide animal ethics committee.

Mice were fed the AIN-93G mouse feed purchased from Specialty Feeds (Western Australia) from time of weaning until 3 months of age to ensure adequate growth and cognitive development. At 3 months of age, mice were randomly separated into three diet groups: low Ω-3:Ω-6, medium Ω-3:Ω-6, and high Ω-3:Ω-6. The low Ω-3:Ω-6 diet incorporated 0.6 g/kg Ω-3, with a 1:29 ratio of Ω-3:Ω-6, representing a similar Ω-3:Ω-6 ratio to current western diets (Blasbalg et al., 2011). The medium Ω-3:Ω-6 diet incorporated 4.2 g/kg Ω-3 with a 1:3.6 ratio of Ω-3:Ω-6, approximately similar to estimated human consumption in the year 1909 (1:6.7) (Blasbalg et al., 2011) and the estimated traditional evolutionary Ω-3:Ω-6 ratio (1:1) (Simopoulos, 2011), which is predicted to be more beneficial than the current intake levels (Simopoulos, 2011). The high Ω-3:Ω-6 diet incorporated 14.2 g/kg Ω-3 with a 2:1 ratio of Ω-3:Ω-6, a level of supplementation beyond what could reasonably be achieved through modifying dietary intake to a more traditional composition (Simopoulos, 2011). For an overview of the fat content of each diet refer to Table 1. Modified diets were based on the AIN-93M adult mouse diet with 5% total fat and only differed in proportion of dietary fatty acids. The AIN-93G and AIN-93M mouse diets are formulations of feed designed for optimal animal health, with a known composition (Reeves et al., 1993). Diet was stored as dry pellets in a sealed vacuum container until required. Once opened, feed was stored at 4°C. A full analysis of the composition of each diet is available in the supplementary information. The medium Ω3:Ω6 diet had fatty acid levels similar to those found in normal rodent chow used in the animal facility (Meat Free Rat and Mouse Diet (Speciality Feeds): total fat content 5%, total *n* − 30.37%, total *n*− 61.31%, with a CV of 43% and 6% respectively). Mice were fed the modified diets for 4 months. A total of 30 mice [WT: low Ω-3:Ω-6 *n* = 10 (7 male + 3 female), medium Ω-3:Ω-6 *n* = 10 (6 male + 4 female), high Ω-3:Ω-6 *n* = 10 (5 male + 5 female)], aged 7 months (middle age), were used for behavioral analyses. There were no differences in outcomes observed between male and female mice in any of the treatment groups. Due to the loose consistency of the feed pellets, many of which disintegrated and fell into the cage, we were unable to measure food intake.

**Table 1:** **Composition of fatty acids in experimental diets (% weight of total fatty acids)**.

**Diets**	**Low Ω-3:Ω-6**	**Med. Ω-3:Ω-6**	**High Ω-3:Ω-6**
Total Ω-6	1.76	1.52	0.70
ARA	trace	0.02	0.07
LA	1.75	1.48	0.56
Total Ω-3	0.06	0.42	1.42
ALA	0.04	0.03	0.02
EPA	Trace	0.06	0.23
DHA	0.04	0.28	1.03
MUFA	1.30	1.27	1.17
PUFA	1.82	1.95	2.14
SFA	1.69	1.59	1.40
Ω-3:Ω-6	1:29	1:3.6	2:1

*Composition of fatty acids in each diet, as a percentage of weight in the overall diet. Ω-3 and Ω-6 PUFA concentrations were achieved through altering quantities of clarified butter, maize oil and Numega Tuna oil. ARA, Arachidonic acid; LA, linolenic acid; ALA, alpha-Linolenic acid; EPA, Eicosapentaenoic acid; DHA, Docosahexaenoic acid; MUFA, monounsaturated fatty acid; PUFA, polyunsaturated fatty acid; SFA, saturated fatty acid*.

### Assessment of cognition-like behavior

#### Open field

The open field test was used to assess general locomotor activity and anxiety like behavior. It consists of a 40 × 40 cm box with black walls 35 cm high into which each mouse was placed for 5 min while behavior was recorded according to published protocols (Baune et al., 2008; Gould et al., 2009; Hart et al., 2010). ANYMAZE recording software (v 4.98) was used to record total distance traveled and time spent in center.

#### Barnes maze

The Barnes Maze was used to assess spatial learning and memory. It consists of an elevated circular table (91 cm), 90 cm above the ground, with 20 circular holes evenly distributed around the periphery of the table. One hole lead to an escape box of sufficient size to house the mouse comfortably while the remaining 19 holes lead to false boxes of insufficient size to house the mouse. Bright overhead lighting provided incentive for the mouse to escape the exposed circular platform by locating the escape box. Barnes Maze procedures were carried out according to published protocols (McLay et al., 1998; Baune et al., 2008; McAfoose et al., 2009). Mice underwent 4 days of training during which each mouse was placed in the center of the apparatus under a cover which was removed at the beginning of the trial. If the escape hole was not located within 3 min, the mouse was gently directed toward the escape hole, followed by relocation to the home cage. The apparatus was cleaned with 70% ethanol between each trial to eliminate olfactory cues. Each mouse received 3 trials per day separated by 15 min. The average latency of the three trials was recorded. On day 5 two probe trials were given for 3 min each. The table was rotated so that the escape box was 90° (probe trial 1) and 180° (probe trial 2) from the original training position. Rotating the table and position of the hole should counteract any olfactory cues used to find the old location, as in a traditional probe trial with no hole. Time to find the old location of the escape box and time to find the new location were recorded. Latency to find the new location gives an indication that the mice learns the escape box has moved and tries to find it in a new position. Probe trial 2 should act as a type of extinction trial, where mice should no longer go straight to the old location (data is not shown). All movements, including latency to find the escape box were recorded using ANYMAZE software.

### Molecular analyses

#### Tissue processing

Each mouse was injected with pentobarbitone (60 mg/kg). Adequate anesthesia was determined by the absence of limb and corneal reflexes. Blood was collected via cardiac puncture from half of the mice after which their brains were retrieved, fresh frozen in liquid nitrogen and stored at −80°C for future protein analysis (WT: low Ω-3:Ω-6 *n* = 5, medium Ω-3:Ω-6 *n* = 5, high Ω-3:Ω-6 *n* = 5). The remaining halves of each treatment group were prepared for immunohistochemistry (IHC). These mice were perfused with a 10 ml of 10% formalin solution through the left ventricle followed by retrieval of brain tissue and storage in 10% formalin (low Ω-3:Ω-6 *n* = 5, medium Ω-3:Ω-6 *n* = 5, high Ω-3:Ω-6 *n* = 5).

#### Serum cytokine quantification

Blood was centrifuged at 2500 rpm for 10 min, at least 30 min following collection, and serum was collected. Serum cytokine levels were measured using the BD Biosciences Cytometric Bead Array (CBA) Mouse Inflammation Kit for the cytokines interleukin (IL)-6, IL-10, monocyte chemoattractant protein (MCP)-1, interferon (IFN)-γ, TNF-α, and IL-12p70 according to the manufacturer's instructions. The theoretical lower detection per cytokine was; 5 pg/ml for IL-6, 17 pg/ml for IL-10, 52.7 pg/ml for MCP-1, 2.5 pg/ml for IFN-γ, 7.3 pg/ml for TNF-α and 10.7 pg/ml for IL-12p70. The Aria (Beckman Coulter) was used to acquire 3000 events for each sample. Individual cytokine concentrations were identified by their fluorescent intensities (Fl-2) and were computed using the individual cytokine standard reference curve using the FCAP Array v3 software (BD Biosciences).

#### Immunohistochemistry

Brains were embedded in paraffin prior to sectioning, with a series of nine 5 μm sections collected at 200 μm intervals from Bregma −1.3 to −3.1, allowing a comprehensive analysis of the hippocampus. Immunohistochemistry was then performed to examine levels of TNF-α (R&D: AF410-NA), doublecortin (DCX) (Millipore: AB2293), Ki67 (Abcam: AB15580), GFAP (Novocastra: NCL:GFAP:GA5), IBA-1 (Santa-Cruz: 28528), caspase-3 (Biovision: 3138-100), and oxo8dG/oxo8G (QED Bioscience 12501). Following de-waxing and dehydration, endogenous peroxidase activity was blocked by incubation with 0.5% hydrogen peroxide in methanol for 30 min. Slides were then washed 2 × 3 min in phosphate buffered saline (PBS) before antigen retrieval by heating at close to boiling point for 10 min (TRIS for doublecortin, citrate for others). Once the slides had cooled below 40°C they were washed with PBS before being blocked with 3% normal horse serum in PBS for 30 min. The appropriate primary antibody was applied to the slides which were left to incubate at room temperature overnight (TNF-α 1:300, IBA-1 1:1000, caspase-3 1:1000, Ki67 1:2000, doublecortin 1:8000, GFAP 1:40000, oxo8dG/oxo8G 1:8000).

The next day slides were washed in 2 × 3 min of PBS before the appropriate species of IgG biotintylated antibody was added for 30 min (Dako). After another PBS wash, slides were incubated with streptavidin peroxidase conjugate for 60 min followed by another rinse with PBS. The immunocomplex was then visualized with precipitation of DAB (Sigma D-5637) in the presence of hydrogen peroxide. Slides were washed to remove excess DAB and lightly counterstained with haematoxylin, dehydrated and mounted with DePeX from histolene.

Slides were subsequently digitally scanned using the Hamamatsu NanoZoomer and examined using the associated NDP.view2 software (Hamamatsu). Sequential images were captured of the hippocampus consisting of the cornu ammonis, dentate gyrus and hippocampal formation and exported for manual cell counting using the inbuilt software within Image J 1.46 (NIH). The number of positive cells were then counted at 40× magnification within each hippocampus (or dentate gyrus alone depending on the immunohistochemical stain) of each slide. For the stains TNF-α, anti-caspase 3, Ki67, and DCX, all positive cells in the relevant brain areas were counted and recorded through the use of the cell tracking tool provided with the software. For GFAP and IBA-1, the image was overlaid with 120 × 120 μm grids, and every second grid for IBA-1 or 10 consistent representative grids for GFAP were counted with the total being corrected for the total area to determine the number of immunopositive cells. The size of the hippocampus or dentate gyrus was then measured within the NDP view software to allowing the final measurement to be expressed as cells/mm^2^. For oxidative stress, the dentate gyrus region from each mouse was ranked on a scale from 0 to 3, with 0 = no staining, 1 = light staining, 2 = moderate staining and 3 = strong staining. The researcher was blinded to the study group of the slides during the slide analysis process. The Coefficient of error was calculated by comparing the total number of immunopositive cells for each subject within each diet and stain to identify the mean and standard error of the mean. The Coefficient of error then was the result of the SEM divided by the Mean. For hippocampal TNFα, the CE for the for high Ω-3:Ω-6 mice was 0.06, for medium Ω-3:Ω-6 mice was 0.12 and for low Ω-3:Ω-6 was 0.10. For TNF-α in the dentate gyrus, the CE was 0.29, 0.21, and 0.31 for high, medium and low Ω-3:Ω-6 mice, respectively. For DCX the CE values were 0.17, 0.18, and 0.05 for high, medium and low Ω-3:Ω-6 mice, respectively. For Ki67 the CE values were 0.17, 0.17, and 0.19 for high, medium and low Ω-3-Ω-6 mice. For GFAP the CE values were 0.04, 0.12, and 0.04 for high medium and low Ω-3:Ω-6 mice. For IBA-1, CE values were 0.07, 0.06, and 0.08 for high, medium and low Ω-3:Ω-6 mice and for caspase-3 CE values were 0.20, 0.17, and 0.17 for high, medium and low Ω-3:Ω-6 mice, respectively.

#### Free fatty acid analyses

Analyses of free fatty acids were carried out by the Waite Analytical Services. Briefly the standardized protocol extracted total lipids from thawed hippocampi tissue samples utilizing a modified Bligh and Dyer method with the addition of heptadecanoic acid as an internal standard (Tu et al., 2013). Free fatty acids were separated via thin layer chromatography and methylated and then analyzed via gas chromatography. The resultant fatty acid methyl esters were identified based on the retention time of standards obtained by Nucheck Prep Inc. (Elysian MN) using the Hewlett-Packard Chemstation data system. Free fatty acids were quantified as a percentage of hippocampal tissue sample.

### Statistical analyses

All data was analyzed using Graphpad PRISM software (version 6.02). Data was tested for Gaussian distribution using the D'Agostino-Pearson omnibus test. All data passed Normality testing. For Barnes maze learning analysis repeated measures Two-Way ANOVA with Tukey's multiple comparisons test was used, to show whether groups showed significant learning or not. For all other data, comparisons of the effect of different diets within mouse strains were tested using One-Way ANOVA with Tukey's *post-hoc* test comparisons. For all results, a *p*-value of < 0.05 was considered significant. All data is represented as mean values ± SEM.

## Results

### The effect of Ω-3:Ω-6 supplementation on weight and general open field measures

Mice were weighed immediately prior to euthanasia. Mice in the low Ω-3:Ω-6 group (28.5 ± 0.95 g) were found to be significantly lighter than medium Ω-3:Ω-6 (35.7 ± 1.78 g) and high Ω-3:Ω-6 diet (34.4 ± 1.73 g) mice (One-Way ANOVA *F* = 6.28, *p* = 0.006, Tukey's *post-hoc* test *p* = 0.007, *p* = 0.029, respectively). There was however no significant correlation between weight and anxiety or weight and immunohistochemistry stain count when analyzed using Pearson correlation.

**Figure 1:**
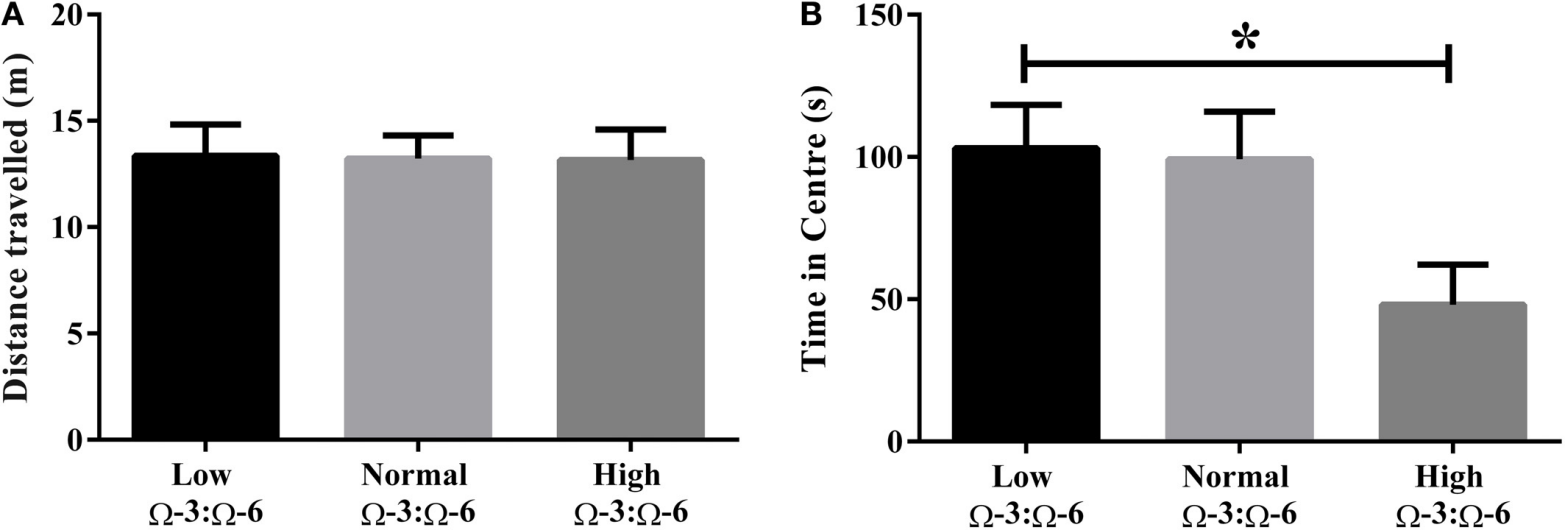
**Locomotor activity and anxiety in the open field. (A)** Locomotor activity of mice in the open field measured by distance traveled and **(B)** anxiety-like behavior in mice measured by time spent in the center of the open field. Data analyzed by One-Way ANOVA within strains with Tukey's *post-hoc* test, ^*^p < 0.05, as indicated on figure. All data presented as mean ± SEM (*n* = 10/group).

Differences in locomotor activity were investigated by recording the distance traveled during the Open Field test. No locomotor differences were observed between low, medium or high Ω-3:Ω-6 diet groups (One-Way ANOVA *F* = 0.14, *p* = 0.87) (Figure 1A).

Anxiety-like behavior was measured in the open field test whereby time spent in the center of the field represents reduced anxiety-like behavior (Flint, 2003; Hart et al., 2010). One-Way ANOVA (*F* = 3.97, *p* = 0.032) with Tukey's *post-hoc* test showed that mice in the high Ω-3:Ω-6 diet group spent significantly less time in the center of the open field compared to the low Ω-3:Ω-6 group (*p* = 0.047), with a trend to also spend less time than the medium Ω-3:Ω-6 group (*p* = 0.068). These results show that the high Ω-3:Ω-6 diet leads to increased anxiety in the open field (Figure 1B).

### Investigating effects of Ω-3:Ω6 dietary supplementation on spatial learning

**Figure 2:**
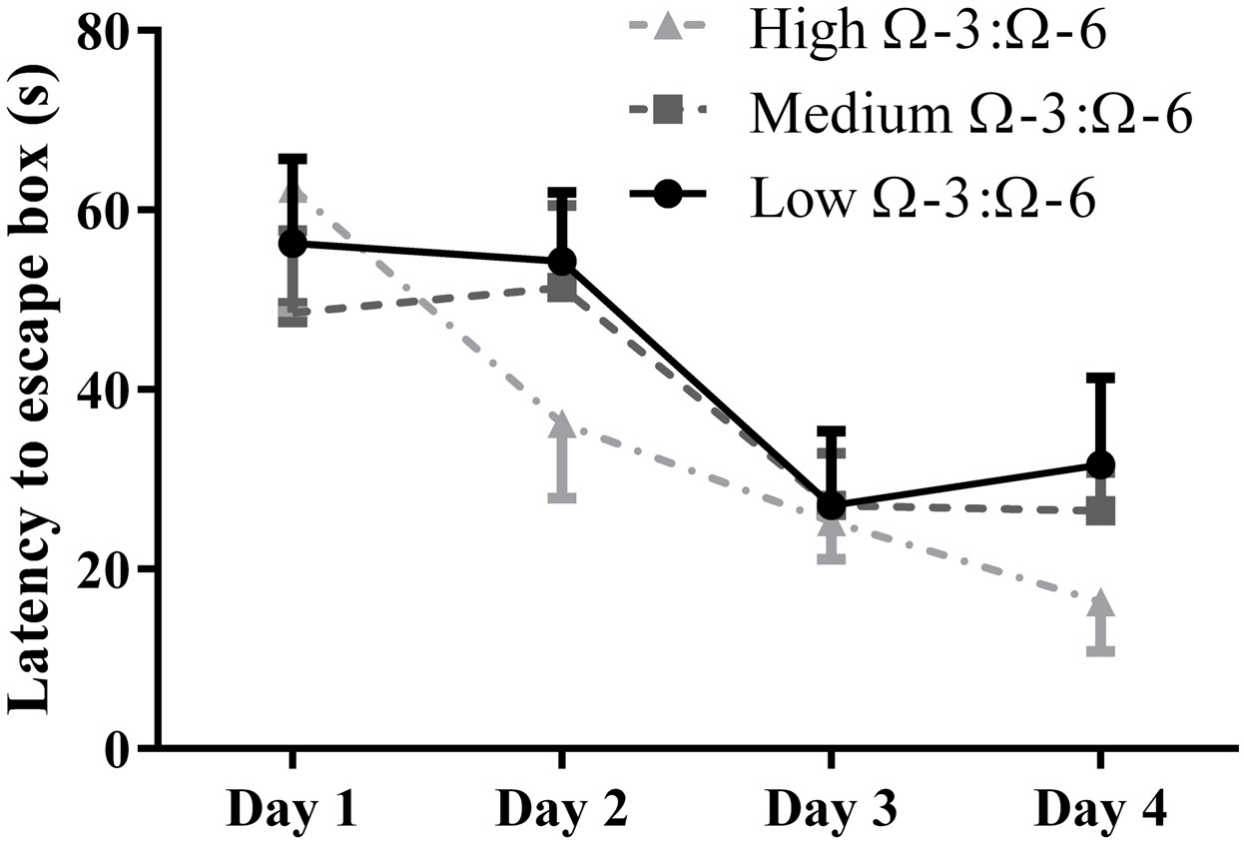
**Learning behavior in the Barnes Maze**. Learning ability of mice on the Barnes Maze measured by latency to the escape location over 4 days of training. All data represent mean ± SEM (*n* = 10/group).

The Barnes Maze was used to investigate learning behavior as described. Learning ability was inferred from reduced latency to locate the escape hole on subsequent training days over 4 days. A repeated measures Two-Way ANOVA was used to analyses latency by Diet and by Day, showing a significant effect of day of training (*F* = 13.0, *p* < 0.0001) but not of diet (*F* = 0.55, *p* = 0.59) or interaction (*F* = 0.85, *p* = 0.54) (Figure 2). Tukey's *post-hoc* comparison of day effect suggests that both the medium and high Ω-3:Ω-6 diet groups showed significant learning over the 4 days, with latency on Day 1 significantly higher than on Day 4 in both the medium (*p* = 0.0047) and high Ω-3:Ω-6 diet groups (*p* = 0.0004). There were no significant differences between latency on any days for mice in the low Ω-3:Ω-6 diet group. These observations suggest that the intake of a high or medium concentration of dietary Ω-3 PUFA's improve the ability of mice to learn across 4 days.

Table 2 shows results of the Probe trial 1, where the escape box was rotated 90° from the old location. One-Way ANOVA showed no difference between diet groups in latency to find the old location (*p* = 0.67, *F* = 0.40) indicating similar spatial memory across all strains, or in time taken to enter the new location (*p* = 0.94, *F* = 0.06).

**Table 2:** **Probe Trial measures of Barnes Maze (retention memory)**.

	**Time to Find old escape box location (s)**	**Time to enter new escape box location (s)**
Low Ω-3:Ω-6	72.3 ± 22.3	136.8 ± 14.3
Medium Ω-3:Ω-6	89.0 ± 24.6	138.8 ± 19.9
High Ω-3:Ω-6	60.3 ± 17.6	130.1 ± 19.3

*Barnes Maze probe trial measures. Data analyzed by One-Way ANOVA. Data represents mean ± SEM (n = 10/group)*.

### Examining effects of Ω-3:Ω-6 supplementation on serum cytokine expression

One-Way ANOVA showed a difference between diet groups in both IFN-γ (*F* = 10.3, *p* = 0.0047) and TNF-α (*F* = 6.81, *p* = 0.01). Tukey's *post-hoc* analysis indicated that both cytokines were increased in the low Ω-3:Ω-6 diet group compared to the medium (IFN-γ *p* = 0.0068, TNF-α *p* = 0.029) and high (IFN-γ *p* = 0.0078, TNF-α *p* = 0.014) diet groups (Table 3). No differences between diet groups were observed for the remaining inflammatory markers (IL-6, IL-10, MCP-1, or IL-12; One-Way ANOVA, all *p* > 0.05).

**Table 3:** **Serum cytokine levels**.

**Cytokines(pg/ml)**	**TNF-α**	**IFN-γ**	**IL-6**	**IL-10**	**IL-12**	**MCP-1**
Low Ω-3:Ω-6	4.59 ± 0.47	5.75 ± 1.87	3.57 ± 1.20	58.1 ± 17.5	20.6 ± 3.79	19.1 ± 4.35
Medium Ω-3:Ω-6	2.65 ± 0.36^*^	0.70 ± 0.27^**^	2.18 ± 0.64	23.8 ± 5.11	12.1 ± 1.51	18.7 ± 2.73
High Ω-3:Ω-6	2.39 ± 0.54^*^	1.03 ± 0.24^**^	2.07 ± 0.29	30.1 ± 6.46	15.4 ± 4.07	18.7 ± 2.46

*Representation of cytokines in serum measured by CBA. Data analyzed by One-Way ANOVA with Tukey's post-hoc test*.

* *p < 0.05*,

** *p < 0.01 C.F. low Ω-3 diet. Data represents mean ± SEM (n = 5/group)*.

### Effects of Ω-3:Ω-6 supplementation on expression of the pro-inflammatory cytokine TNF-α in the hippocampus

**Figure 3:**
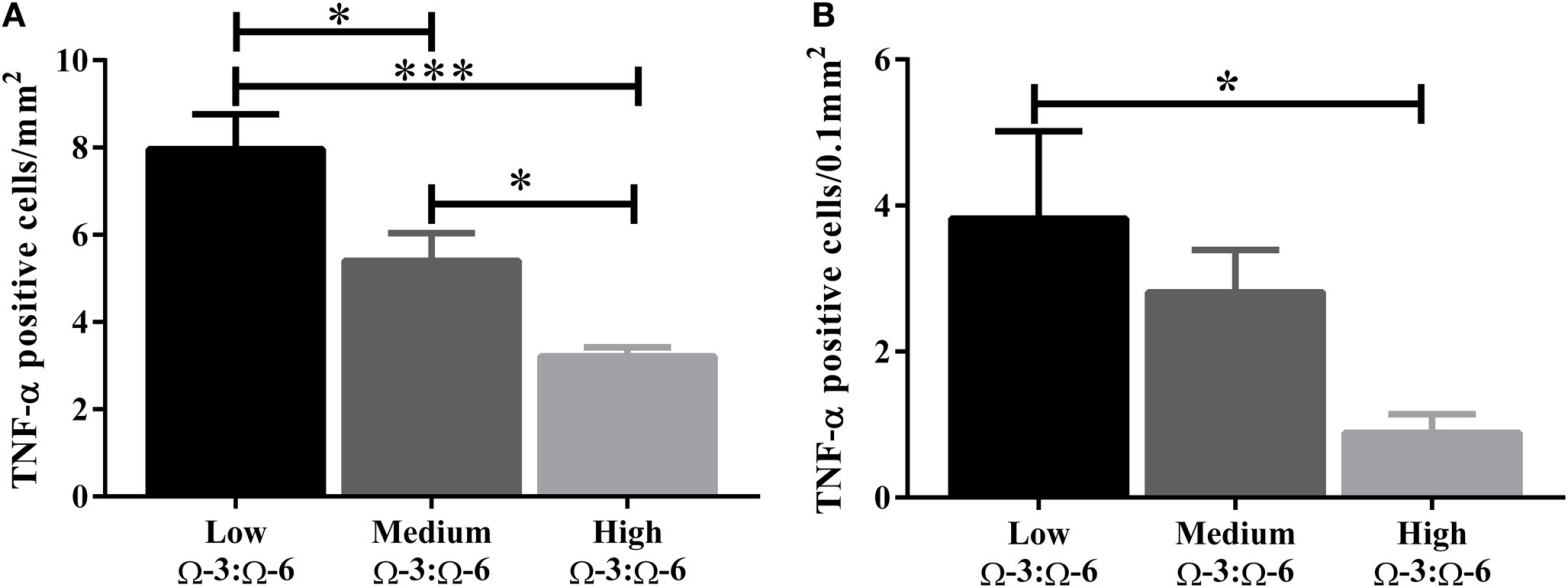
**Total number of TNF-α positive cells in the hippocampus**. The total number of TNF-α positive cells in **(A)** the hippocampus (including the dentate gyrus) and **(B)** the dentate gyrus only. Data analyzed by One-Way ANOVA within strains with Tukey's *post-hoc* test, ^*^p < 0.05, ^***^p < 0.001, as indicated on figure. All data presented as mean ± SEM (*n* = 5/group).

**Figure 4:**
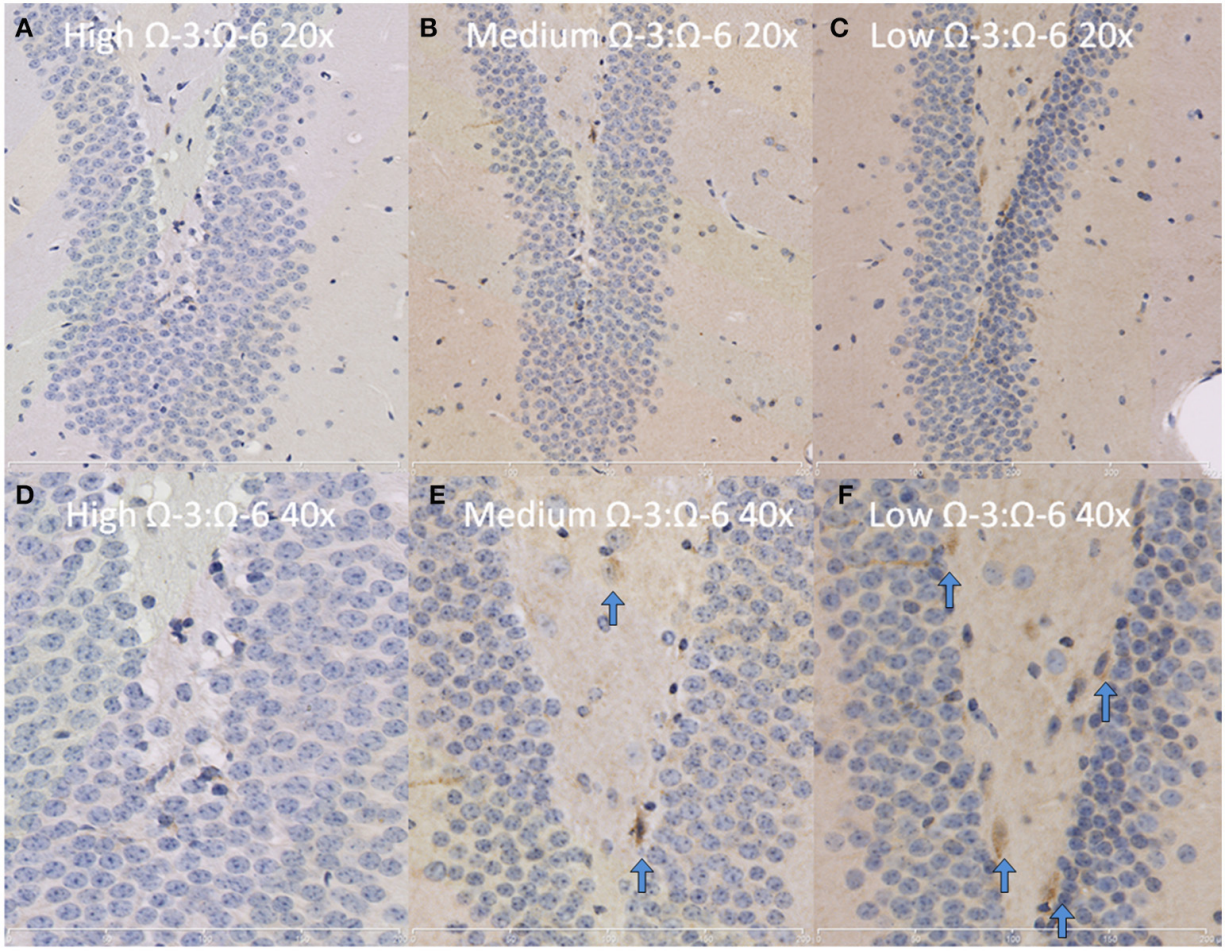
**Representative images of TNF-α expression in the dentate gyrus**. Representative images of the hippocampus centered on the dentate gyrus to demonstrate expression of TNF-α positive cells at 20× magnification in **(A)** High Ω-3:Ω-6 mice, **(B)** medium Ω-3:Ω-6 mice, **(C)** low Ω-3:Ω-6 mice and at 40× magnification in **(D–F)**, respectively. Arrows signify relevant stained cells.

The hippocampus (including the cornu ammonis, dentate gyrus and hippocampal formation) was investigated due to its' role in spatial learning and memory (Jarrard, 1993; Rolls and Kesner, 2006; Neves et al., 2008). Within the hippocampus, the dentate gyrus was also investigated due to its role in memory and in neurogenesis (Neves et al., 2008). The cytokine TNF-α was investigated due to its' critical role in learning and memory. Recent evidence suggests that a basal level of TNF-α is required for normal memory and learning however excessive levels may be detrimental (Baune et al., 2008; McAfoose and Baune, 2009; Camara et al., 2013). Hippocampal TNF-α positive cells were reduced in a dose dependent manner as shown by less TNF- α positive cells with increasing Ω-3 dietary concentration (One-Way ANOVA *F* = 16.55, *p* < 0.001; Tukeys' *post-hoc* test low Ω-3:Ω-6 vs. medium Ω-3:Ω-6 *p* = 0.025, low Ω-3:Ω-6 vs. high Ω-3:Ω-6 *p* = 0.0003, medium Ω-3:Ω-6 vs. high Ω-3:Ω-6 *p* = 0.041) (Figure 3A). Furthermore, when the dentate gyrus was examined separately, the high Ω-3:Ω-6 diet mice had significantly less TNF-α expression than low Ω-3:Ω-6 diet mice (One-Way ANOVA *F* = 4.45, *p* = 0.038; Tukeys' *post-hoc* test *p* = 0.036) (Figure 3B). A representative image of TNF-α expression in the dentate gyrus is depicted in Figure 4. These results suggest that higher Ω-3:Ω-6 dietary ratio's reduced the expression of the pro-inflammatory cytokine TNF-α in the hippocampus and the dentate gyrus.

### Effects of Ω-3:Ω-6 supplementation on neuronal progenitor proliferation and gliogenesis markers in the dentate gyrus

To investigate the effect of Ω-3 on neuronal progenitor proliferation, KI67 (proliferating cells) and DCX (immature neurons) expression in the dentate gyrus were examined by staining hippocampal slices with anti-Ki67 antibodies and anti-DCX antibodies. Gliogenesis in the hippocampus was investigated by staining of hippocampal slices with anti-GFAP (astrocyte marker) and anti-IBA-1 (microglia marker) antibodies. The subsequent slides were digitally imaged followed by manual examination.

**Figure 5:**
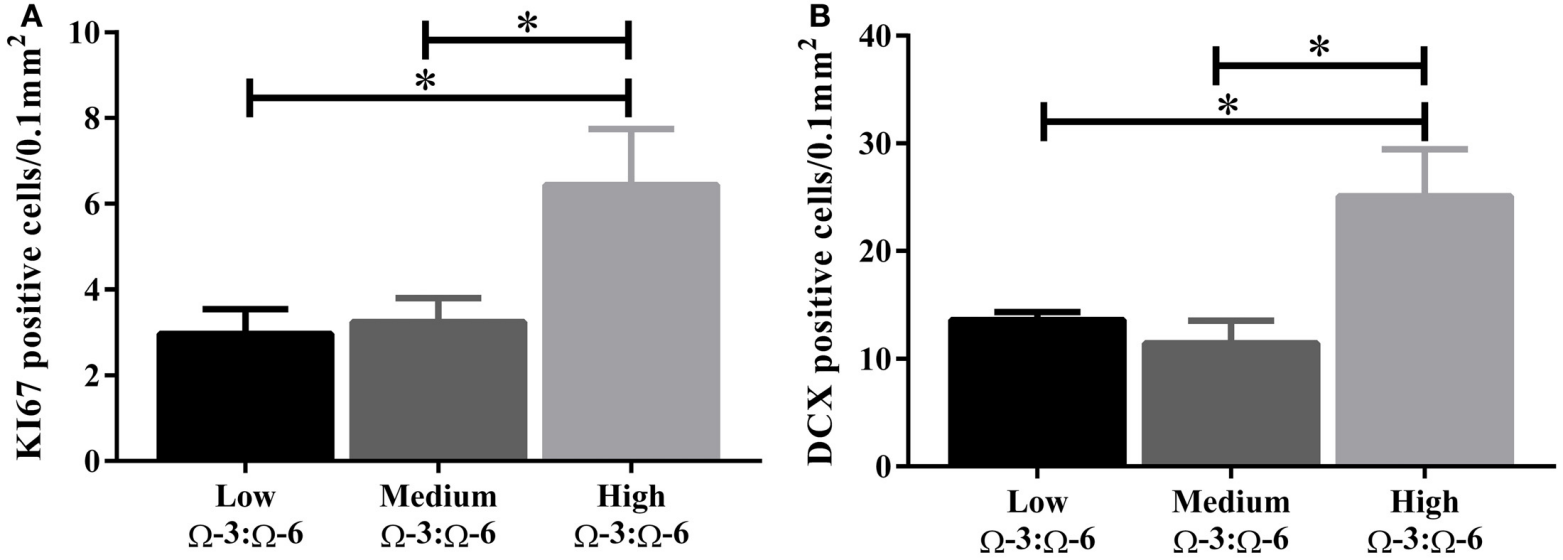
**Effect of Ω-3:Ω-6 diet on neurogenesis in the dentate gyrus**. Number of KI67 positive cells **(A)** and DCX positive cells **(B)** in the dentate gyrus. Data analyzed by One-Way ANOVA within strains with Tukey's *post-hoc* test, ^*^p < 0.05 as indicated on figure. All data represented as mean ± SEM (*n* = 5/group).

**Figure 6:**
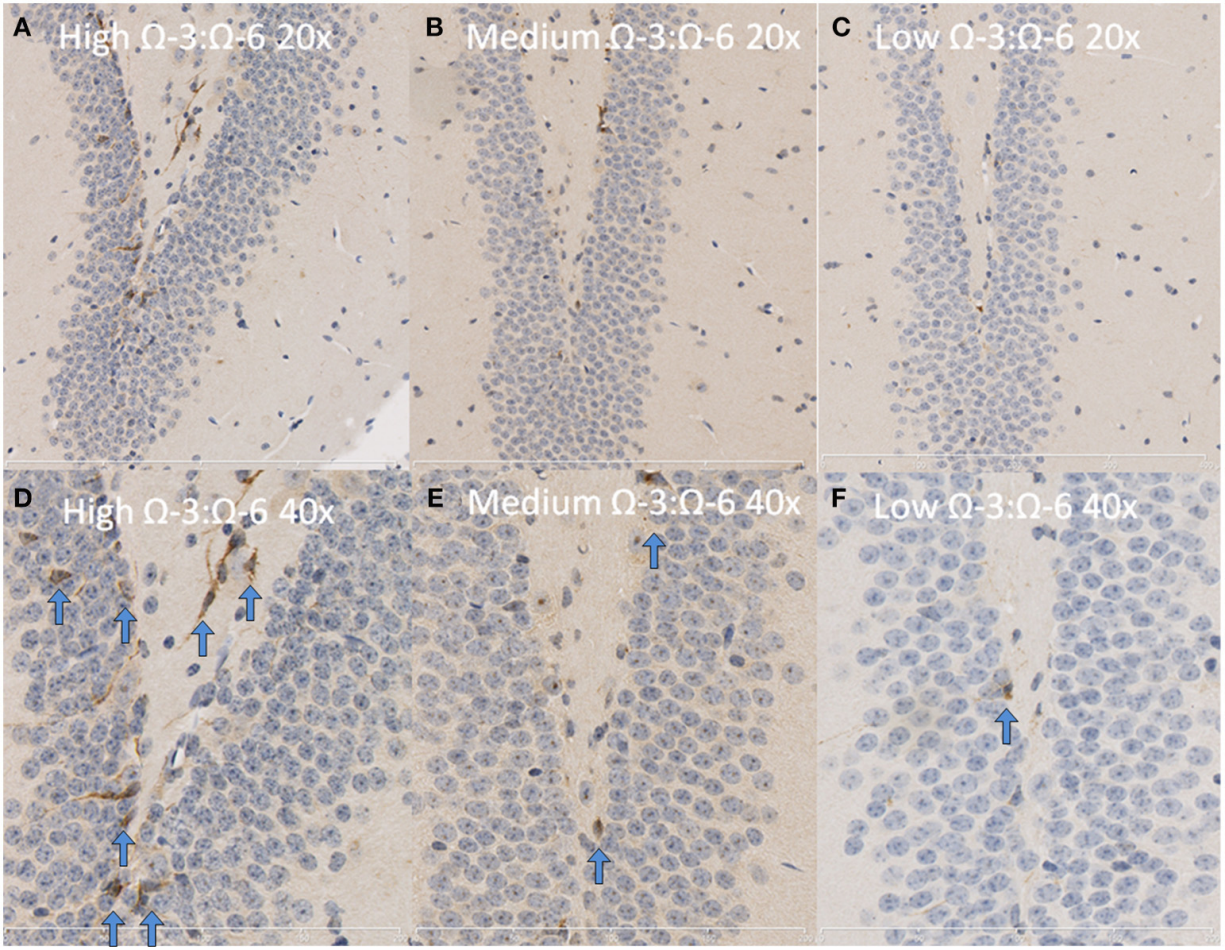
**Representative images of DCX expression in the dentate gyrus**. Representative images of the hippocampus centered on the dentate gyrus to demonstrate expression of DCX positive cells at 20× magnification in **(A)** high Ω-3:Ω-6 mice, **(B)** medium Ω-3:Ω-6 mice, **(C)** low Ω-3:Ω-6 mice and at 40× magnification in **(D–F)**, respectively. Arrows signify relevant stained cells.

**Figure 7:**
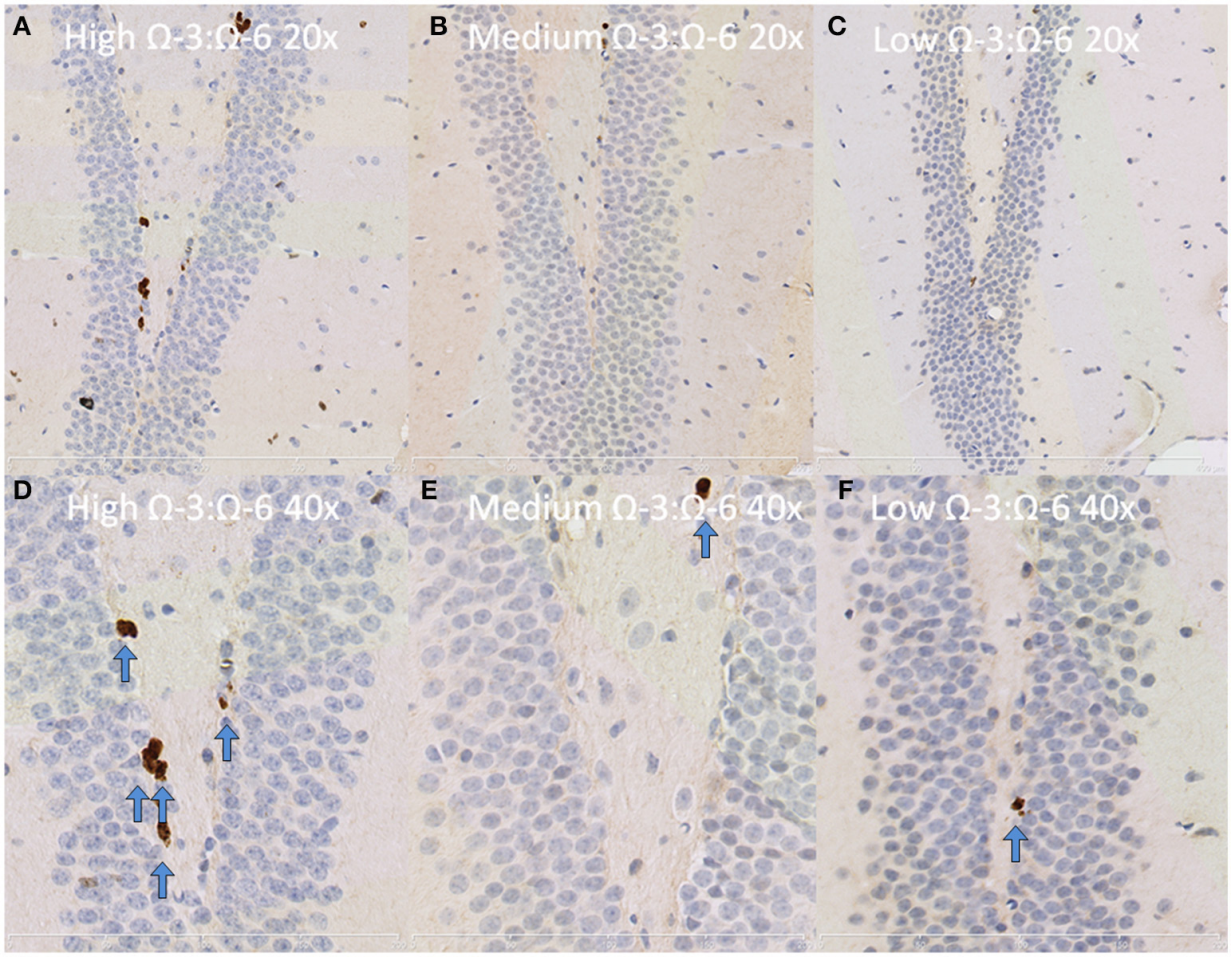
**Representative images of Ki67 expression in the dentate gyrus**. Representative images of the hippocampus centered on the dentate gyrus to demonstrate expression of Ki67 positive cells at 20× magnification in **(A)** high Ω-3:Ω-6 mice, **(B)** medium Ω-3:Ω-6 mice, **(C)** low Ω-3:Ω-6 mice and at 40× magnification in **(D–F)**, respectively. Arrows signify relevant stained cells.

Under physiological conditions, the high Ω-3:Ω-6 diet group showed a significant increase in Ki67 and DCX positive cells in the dentate gyrus compared to the low and medium Ω-3:Ω-6 diet groups (Ki67: One-Way ANOVA *F* = 5.21 *p* = 0.026; Tukeys' *post-hoc* test *p* = 0.030, *p* = 0.048, respectively; DCX One-Way ANOVA *F* = 6.70, *p* = 0.011; Tukeys' *post-hoc* test *p* = 0.0.035, *p* = 0.013, respectively) (Figures 5A,B). The representative images of these stains can be viewed in Figures 6, 7. In contrast, dietary Ω-3:Ω-6 ratio content was found not to modify microglia or astrocyte number in the hippocampus [One-Way ANOVA microglia (IBA-1) *F* = 2.40, *p* = 0.13, astrocytes (GFAP) *F* = 0.20, *p* = 0.82] (representative images can be seen in Supplementary Figures 1, 2). This indicates that the increase in cell proliferation in high Ω-3:Ω-6 mice was not associated with an increase in astrocyte or microglial number.

### Effects of Ω-3:Ω-6 supplementation on oxidative stress and cell death in the dentate gyrus

**Figure 8:**
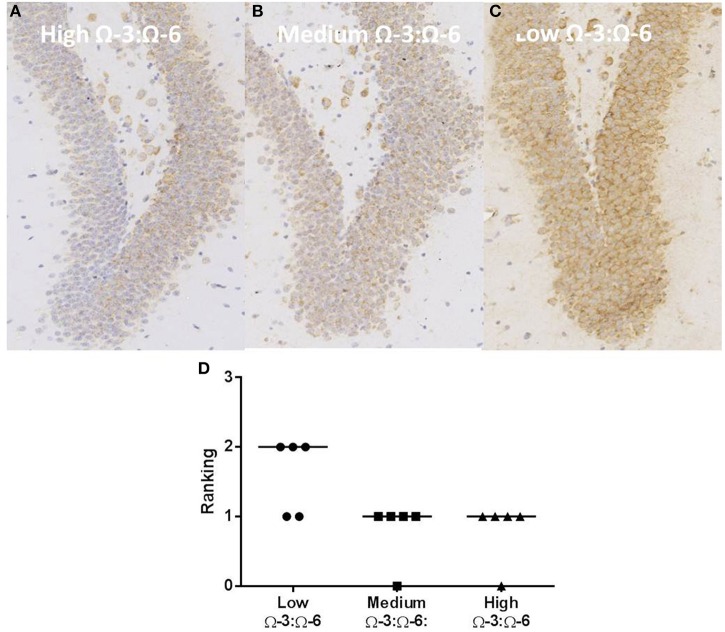
**Effect of Ω-3:Ω-6 diet on oxidative stress in the dentate gyrus**. Representative images of oxo8dG/oxo8G expression in the dentate gyrus at 20× magnification in **(A)** high Ω-3:Ω-6 mice, **(B)** medium Ω-3:Ω-6 mice, **(C)** low Ω-3:Ω-6 mice, with representation of the median intensity of staining in each group **(D)**.

Oxidative stress was investigated through staining hippocampal slices with an antibody to oxo8dG/oxo8G which measures DNA/RNA oxidative damage. Dietary Ω-3:Ω-6 ratio modified the level of oxidative stress, with a significant difference between the median of each group (Kruskal Wallis *p* < 0.05), with the low Ω-3:Ω-6 diet having a median of 2, representing moderate levels of staining compared to a median of 1, indicating a low level of staining in the medium and high Ω-3:Ω-6 diet groups (Figure 8).

Similarly, cell death was investigated through staining hippocampal slices with anti-caspase 3 antibodies (a protein involved in cell apoptosis) and examination of digital images. The dentate gyrus was investigated to determine if the increase in cell proliferation and immature neurons was associated with an increase in cell death. Dietary Ω-3:Ω-6 ratio was not found to modify cell death in the dentate gyrus (One-Way ANOVA *F* = 0.54, *p* = 0.59) (representative images can be viewed in Supplementary Figure 3).

### Free fatty acid analysis of the hippocampus

Analysis of Ω-3 and Ω-6 concentrations in the hippocampus of test mice confirmed that alterations in dietary levels of PUFAs do translate to the brain and that mice did consume enough of the pellets to see a change (Table 4). Concentration of Ω-3 increased from the low to high Ω-3:Ω-6 groups (One-Way ANOVA *p* = 0.0031) and Tukey's *post-hoc* test showed that the low Ω-3:Ω-6 group had significantly lower levels of Ω-3 in the hippocampus than both the medium (*p* = 0.037) and high Ω-3:Ω-6 groups (*p* = 0.0027). Concentration of Ω-6 also decreased from the low to high Ω-3:Ω-6 groups (One-Way ANOVA *p* = 0.0003) and Tukey's *post-hoc* test showed that the high Ω-3:Ω-6 group had significantly lower levels of Ω-6 in the hippocampus than both the medium (*p* = 0.0022) and low Ω-3:Ω-6 groups (*p* = 0.0004). Analysis of Ω-3:Ω-6 ratio in the hippocampus also confirmed the expected differences based on diet. One-Way ANOVA (*p* < 0.0001) showed that the low Ω-3:Ω-6 group had a significantly lower ratio than both the medium (*p* = 0.026) and high Ω-3:Ω-6 diets groups (*p* < 0.0001) while the medium Ω-3:Ω-6 diet group also showed a significantly lower ratio than the high Ω-3:Ω-6 diet group (*p* = 0.0022).

**Table 4:** **Hippocampus PUFA levels**.

	**Ω-3 (% Free fatty acids)**	**Ω-6 (% Free fatty acids)**	**Ω-3:Ω-6**
Low Ω-3:Ω-6	10.2 ± 0.61	22.7 ± 0.78	0.45 ± 0.01
Medium Ω-3:Ω-6	12.7 ± 0.62^*^	22.1 ± 0.71	0.58 ± 0.03^*^
High Ω-3:Ω-6	13.8 ± 0.54^**^	18.0 ± 0.25^***^^,^^##^	0.77 ± 0.04^****^^,^^##^

Representation of Ω-3 Ω-6 PUFA levels measured in hippocampus. Data analyzed by One-Way ANOVA with Tukey's post-hoc test. ^*^C.F. low Ω-3 diet,

## *C.F. medium Ω-3:Ω-6 diet*.

* *p < 0.05*,

** *p < 0.01*,

*** *p < 0.001*,

**** *p < 0.0001. Data represents mean ± SEM (n = 5/group)*.

## Discussion

This study demonstrated that long-term supplementation and increasing the dietary ratio of Ω-3:Ω-6 PUFA in unchallenged mature adult (7 month old) WT mice leads to increased anxiety and improved hippocampal dependent spatial memory, with increases in neuronal progenitor proliferation and decreases in TNF-α expression, as well as oxidative stress, within the hippocampus. The effects on behavior, TNF-α expression and neuronal progenitor proliferation appeared to be dose dependent, with the high Ω-3:Ω-6 diet group demonstrating the highest levels of anxiety and significant learning in the Barnes Maze, the lowest number of TNF-α positive cells within the hippocampus, and the highest rate of neurogenesis whilst the low Ω-3:Ω-6 diet group had the worst performance on the Barnes Maze, the highest TNF-α expression and the highest levels of oxidative stress. This suggests that Ω-3 diet and Ω-3:Ω-6 ratios may play an important role in maintaining normal behavior throughout life.

The three different doses of Ω-3 supplementation used in this study were designed to represent relevant human equivalent quantities of Ω-3 and Ω-6 consumption. The low Ω-3:Ω-6 diet corresponds to a typical western diet, the medium Ω-3:Ω-6 diet represents a physiologically traditional diet, while the high Ω-3:Ω-6 diet represents considerably higher supplementation again (Blasbalg et al., 2011; Simopoulos, 2011). As previously discussed, increasing dietary Ω-3 increases the Ω-3:Ω-6 ratio, allowing for higher concentrations of Ω-3 derived metabolites to form, which are considerably less pro-inflammatory than their Ω-6 derived counterparts (Wall et al., 2010). The use of specially designed lab diets based on the AIN-93 M standard was done to eliminate the inherent variability in standard lab chow diets. It should be noted that for the first 2 months after weaning, mice were fed with a standard diet (AIN- 93G) designed to support a healthy development. Any effect of dietary Ω-3:Ω-6 supplementation thereafter can be attributed to the effect of Ω-3 supplementation and Ω-6 reduction on adult central nervous system function, and not due to Ω-3 deficiency during neurodevelopment, which has been previously documented (Gibson et al., 2001; Salem et al., 2001; Ryan et al., 2010; Bhatia et al., 2011; Rombaldi Bernardi et al., 2012). Furthermore, our modified diets were given for 4 months (~60% of lifespan), where supplementation >10% of lifespan has previously been suggested to be beneficial (Hooijmans et al., 2012). Previous shorter-term studies have demonstrated that 8 weeks of high Ω-3 diet supplementation attenuated age related increases in TNF-α, IL-6 and IL-1β and improved spatial memory as compared to an Ω-3 deficient diet (Labrousse et al., 2012). No previous studies using a treatment as long as this one in unchallenged aged mice have been performed. The longer-term supplementation given in this study directs focus on the preventative actions of a high Ω-3-Ω-6 PUFA diet ratio in healthy, unchallenged mice up to middle age during early adulthood. Our results represent evidence for a preventative action of a high Ω-3:Ω-6 ratio against cognitive decline associated with early aging. It is important to note that higher dietary Ω-3:Ω-6 dietary concentrations resulted in increased hippocampal Ω-3:Ω-6 concentrations. This confirms that mice consumed as adequate amount of each diet, and that dietary PUFA modulated brain PUFA concentration thus providing an explanation for the central effects of ingested fatty acids. We did note that the mice consuming the medium and high Ω-3:Ω-6 PUFA diet were significantly heavier than the mice consuming the low Ω-3:Ω-6 diet. Previous reviews have linked obesity to higher levels of pro-inflammatory cytokines (Lumeng and Saltiel, 2011), and thus, as the higher Ω-3:Ω-6 PUFA diets resulted in reduced inflammation, the weight of mice is unlikely to have played a role.

No differences in locomotor activity were seen in the open field following different diets, however the high Ω-3:Ω-6 PUFA diet led to lower time spent in the center of the open field, suggesting increased anxiety. Previous studies investigating similar parameters have shown varying results following Ω-3 deficiency or supplementation (Fedorova and Salem, 2006). For example, several studies showed no difference between Ω-3 deficient and control mice (Nakashima et al., 1993; Carrié et al., 2000a), or between high, low and normal Ω-3: Ω-6 diet groups, consistent with our study (Wainwright et al., 1997). However, other studies have shown Ω-3 deficient mice to have increased locomotor activity (Umezawa et al., 1995; Raygada et al., 1998; Fedorova and Salem, 2006) or mice given high Ω-3 diets had reduced activity (Rockett et al., 2012), while further studies have shown different effects of diet depending on age (Carrié et al., 2000b). All of these studies have used different methods to measure locomotor activity, as well as different dosing protocols, including the use of different foods to increase or decrease Ω-3 consumption and either giving it directly to the study mice at different ages or giving it to the mother before and during gestation, therefore direct comparison between studies is difficult. Similarly, various studies have shown different effects on anxiety behavior in animals after altering Ω-3:Ω-6 PUFA levels in their diet (Fedorova and Salem, 2006). Ω-3 deficient mice have previously been shown to have everything from decreased anxiety (Nakashima et al., 1993; Francès et al., 1995), increased anxiety (Carrié et al., 2000a; Fedorova and Salem, 2006), or no change in anxiety measured by time spent in the open arm of an elevated plus maze (Belzung et al., 1998; Moriguchi et al., 2000).

Despite the high Ω-3:Ω-6 PUFA diet leading to increased anxiety in the open field, as it has been suggested that anxiety may exacerbate poor memory (Spencer et al., 2010), the Ω-3:Ω-6 PUFA ratio within the diet appears to be important for normal cognition, with only the normal and high Ω-3:Ω-6 diet groups showing normal learning across the 4 days on the Barnes Maze, as shown by a significant decrease in escape latency from day 1 to day 4. High Ω-3:Ω-6 supplementation may act through a variety of mechanisms to support normal cognition. Here, it was found that a low Ω-3:Ω-6 diet was associated with increased oxidative stress within the dentate gyrus, with previous studies demonstrating that oxidative stress contributes to age related impairment in learning and memory (Liu et al., 2003). Furthermore, it was found that a high Ω-3:Ω-6 diet ratio reduces the hippocampal TNF-α expression. Whilst at basal levels cytokines are intimately involved in cognitive processes (McAfoose and Baune, 2009), their overexpression is associated with increased risk of multiple neuropsychiatric disorders including cognitive decline (McAfoose and Baune, 2009). As cognition-like behavior improved in the high Ω-3:Ω-6 group while hippocampal TNF-α including dentate gyrus TNF-α decreased, this may represent a healthier concentration of TNF-α as compared to the low Ω-3:Ω-6 group. Interestingly, this decrease in dentate gyrus TNF-a coincided with increased neuronal progenitor proliferation. In this study, Ω-3 supplementation in conjunction with Ω-6 restriction also reduced systemic TNF-α and IFN-γ concentration. This is likely to predominantly occur through increased Ω-3 displacing Ω-6 from the common metabolic enzyme pathway leading to increased Ω-3 derived prostaglandins, leukotrienes, and thromboxanes which are less pro-inflammatory than their Ω-6 derived counterparts (Tassoni et al., 2008; Farooqui, 2009; Wall et al., 2010). Additionally, cell culture studies have demonstrated that Ω-3 down regulates the p38 MAPK signaling pathway resulting in decreased nitric oxide synthase, decreasing nitric oxide, TNF-α and prostaglandin E2 (Antonietta Ajmone-Cat et al., 2012). Furthermore, Ω-3 promotes the production of peroxisome proliferator-activated receptor (PPAR)-γ, which is known to regulate microglia and astrocyte cytokine production. Lastly, Ω-3 has been demonstrated to reduce pathological microglia and astrocyte activation which occurs with aging (Gupta et al., 2012; Labrousse et al., 2012). The modulation of systemic and central inflammation together appears to promote a neuroprotective environment in these unchallenged mature adult (7 month old) WT mice.

Evidence regarding the actions of Ω-6 suggests that while it is an essential fatty acid and necessary in small amounts for good health, large quantities such as in current western diets have been shown to have a detrimental and pro-inflammatory effect (Wall et al., 2010; Blasbalg et al., 2011; Simopoulos, 2011). Thus, the reduction of Ω-6 PUFA in the high Ω-3:Ω-6 diet may also be contributing to the protective actions of this diet that were observed in our study.

Further potential mechanisms of improved cognition include the promotion of neuronal progenitor proliferation, which was only noted in mice given the high Ω-3:Ω-6 diet. Increased neurogenesis in the subgranular zone of the hippocampus in young mice (Valente et al., 2009), and in cell culture (Antonietta Ajmone-Cat et al., 2012), have previously been shown to occur with Ω-3 supplementation. This is likely to occur through increased BDNF levels (Jiang et al., 2009), while Ω-3 deprivation has previously been shown to decrease BDNF and phosphorylated CMP response element binding protein (pCREB) (Bhatia et al., 2011). This is the first study to demonstrate that a high Ω-3:Ω-6 diet supports adult neurogenesis (as measured by Ki67 and DCX expression in the dentate gyrus). A high Ω-3:Ω-6 dietary ratio has been demonstrated to have these effects in an approximately middle-age (7 months old) mouse model, supporting a preventative action of Ω-3 PUFA against cognitive decline, thus, investigation of long-term supplementation in young-middle aged human populations may be astute.

In contrast to the demonstrated effects on hippocampal TNF-α levels and on neurogenesis, no effect of diet was demonstrated on astrocyte number or microglia number in this hippocampus of this unchallenged mouse model. Current literature suggests that inappropriate microglia activation plays a key degenerative role in the progression of cognitive decline (Antonietta Ajmone-Cat et al., 2012; Czirr and Wyss-Coray, 2012). This study suggests that in an unchallenged mouse model, increased microglia number is not a major contributing factor and other mechanisms such as the promotion of neuronal progenitor proliferation and modulation of cytokine activity may be more significant.

In clinical studies of age related cognitive decline (ARCD) utilizing Ω-3 supplementation, the dose administered varies from 400 mg/day (Geleijnse et al., 2012) to 2800 mg/day (Van De Rest et al., 2008), while doses of up to 9600 mg/day have been trialed in depression. These doses, supplemented to a typical western diet, result in Ω-3:Ω-6 PUFA ratios of slightly less than the medium Ω-3 supplementation diet in this study (Blasbalg et al., 2011). The total fat intake of this study was standardized to 5% making quantification of human equivalent intakes speculative. Fat restriction allows for precise manipulation of the Ω-3:Ω-6 ratio without using large quantities of Ω-3 PUFA and the increased energy content that comes with fat ingestion. Specifically, in a fat restricted diet, relatively smaller increase in Ω-3 content corresponds to a greater change in Ω-3:Ω-6 ratio. Thus, achieving a similar Ω-3:Ω-6 ratio as the high Ω-3 mouse diet would require considerable fat restriction, or a targeted reduction of Ω-6 intake (Blasbalg et al., 2011). It will be necessary to monitor whether increasing the dose in future clinical trials proves more efficacious, although consideration must be made to investigating dietary fat restriction coupled with Ω-3 supplementation.

In conclusion, long-term Ω-3 supplementation coupled with Ω-6 restriction was demonstrated to increase anxiety, improve cognitive function, reduce TNF-α expression and enhance neuronal progenitor proliferation in unchallenged mature adult (7 month old) mice. These results support the view that in translational research, the preventive effects of a long-term high Ω-3:Ω-6 diet in older adults at risk of cognitive decline require further investigation, also addressing the dose required to result in clinically demonstrable preventive effects on cognitive function. It is unclear however whether supplementation such as used in the current study would overcome a long term significantly pro-inflammatory insult. Further trials investigating Ω-3 supplementation in long term, pro-inflammatory conditions may provide further insight into the efficacy of a high Ω-3:Ω-6 diet in such conditions. Future studies should also include a wider range of behavioral tests, to confirm the effects of Ω-3:Ω-6 ratio in diet on anxiety and cognition, at a range of ages up to old age in mice.

## Author contributions

Authors Trent Grundy, Catherine Toben and Bernhard T. Baune designed the study. Author Trent Grundy performed the behavioral experiments, immunohistochemistry and cell analysis, statistical analysis under the supervision of authors Emily J. Jaehne and Frances Corrigan Trent Grundy wrote the first draft of the manuscript. All authors contributed to manuscript revisions and have approved the final manuscript.

### Conflict of interest statement

The authors declare that the research was conducted in the absence of any commercial or financial relationships that could be construed as a potential conflict of interest.
