# Supplementary material for: Long-term omega-3 supplementation modulates behavior, hippocampal fatty acid concentration, neuronal progenitor proliferation and central TNF-α expression in 7 month old unchallenged mice
Source: Front Cell Neurosci. 2014 Nov 21;8:399. doi: 10.3389/fncel.2014.00399 (PMC4240169; doi:10.3389/fncel.2014.00399)
Supplement: Supplementary file 2 [file DataSheet2.PDF]

3150 Great Eastern Hwy
Glen Forrest
Western Australia 6071
p: +61 8 9298 8111
F: +61 8 9298 8700
Email: info@specialtyfeeds.com

**Diet**

# SF12-035 5% Fat Moderate N3 Semi-Pure Modification of AIN93M Rodent Diet

A 5% fat (moderate n3 fat) semi-pure diet formulation for laboratory rats and mice based on AIN-93M. This formulation satisfies the nutritional requirements for maintenance of rats and mice. Some modifications have been made to the original formulation to suit locally available raw materials.

- Fat content has been increased 5%. This results in an increased energy content over the standard diet.
- Clariffied Butter, Maize Oil and Numega Tuna oil has replaced Canola oil.

## Calculated Nutritional Parameters

| | |
|---|---|
| Protein | 13.60% |
| Total Fat | 5.00% |
| Crude Fibre | 4.70% |
| AD Fibre | 4.70% |
| Digestible Energy | 16 MJ / Kg |
| % Total calculated digestible energy from lipids | 11.70% |
| % Total calculated digestible energy from protein | 15.10% |

## Diet Form and Features

- Semi pure diet. 12 mm diameter pellets.
- Pack size 5 Kg, vacuum packed in oxygen impermeable plastic bags, under nitrogen. Bags are packed into cardboard cartons to protect them during transit. Smaller pack quantity on request.
- Diet suitable for irradiation but not suitable for autoclave.
- Lead time 2 weeks for non-irradiation or 4 weeks for irradiation.

## Ingredients

| | |
|---|---|
| Casein (Acid) | 140 g/Kg |
| Sucrose | 100 g/Kg |
| Ghee (Clarified Butter) | 14.4 g/Kg |
| Maize Oil | 24.9 g/Kg |
| Numega Tuna Oil | 10.7 g/Kg |
| Cellulose | 50 g/Kg |
| Wheat Starch | 462 g/Kg |
| Dextrinised Starch | 155 g/Kg |
| DL Methionine | 1.8 g/Kg |
| Calcium Carbonate | 13.1 g/Kg |
| Sodium Chloride | 2.6 g/Kg |
| AIN93 Trace Minerals | 1.4 g/Kg |
| Potassium Citrate | 1.0 g/Kg |
| Potassium Dihydrogen Phosphate | 8.8 g/Kg |
| Potassium Sulphate | 1.6 g/Kg |
| Choline Chloride (75%) | 2.5 g/Kg |
| AIN93 Vitamins | 10 g/Kg |

| Calculated Amino Acids | |
|---|---|
| Valine | 0.88% |
| Leucine | 1.26% |
| Isoleucine | 0.61% |
| Threonine | 0.56% |
| Methionine | 0.56% |
| Cystine | 0.04% |
| Lysine | 1.04% |
| Phenylanine | 0.70% |
| Tyrosine | 0.70% |
| Tryptophan | 0.20% |

| Calculated Total Minerals | |
|---|---|
| Calcium | 0.47% |
| Phosphorous | 0.35% |
| Magnesium | 0.08% |
| Sodium | 0.15% |
| Chloride | 0.16% |
| Potassium | 0.40% |
| Sulphur | 0.23% |
| Iron | 75 mg/Kg |
| Copper | 7.0 mg/Kg |
| Iodine | 0.2 mg/Kg |
| Manganese | 20 mg/Kg |
| Cobalt | No data |
| Zinc | 50 mg/Kg |
| Molybdenum | 0.15 mg/Kg |
| Selenium | 0.3 mg/Kg |
| Cadmium | No data |
| Chromium | 1.0 mg/Kg |
| Fluoride | 1.0 mg/Kg |
| Lithium | 0.1 mg/Kg |
| Boron | 3.4 mg/Kg |
| Nickel | 0.5 mg/Kg |
| Vanadium | 0.1 mg/Kg |

| Calculated Total Vitamins | |
|---|---|
| Vitamin A (Retinol) | 4 110 IU/Kg |
| Vitamin D (Cholecalciferol) | 1 000 IU/Kg |
| Vitamin E (a Tocopherol acetate) | 76 mg/Kg |
| Vitamin K (Menadione) | 1 mg/Kg |
| Vitamin C (Ascorbic acid) | None added |
| Vitamin B1 (Thiamine) | 6 mg/Kg |
| Vitamin B2 (Riboflavin) | 6 mg/Kg |
| Niacin (Nicotinic acid) | 30 mg/Kg |
| Vitamin B6 (Pryridoxine) | 7 mg/Kg |
| Pantothenic Acid | 16 mg/Kg |
| Biotin | 200 ug/Kg |
| Folic Acid | 2 mg/Kg |
| Inositol | None added |
| Vitamin B12 (Cyancobalamin) | 102 ug/Kg |
| Choline | 1 450 mg/Kg |

| Calculated Fatty Acid Composition | |
|---|---|
| Saturated Fat C12:0 and less | 0.13% |
| Myristic Acid 14:0 | 0.21% |
| Palmitic Acid 16:0 | 0.95% |
| Stearic Acid 18:0 | 0.26% |
| Palmitoleic Acid 16:1 | 0.08% |
| Oleic Acid 18:1 | 1.11% |
| Gadoleic Acid 20:1 | Trace |
| Linoleic Acid 18:2 n6 | 1.48% |
| a Linolenic Acid 18:3 n3 | 0.03% |
| Arachadonic Acid 20:4 n6 | 0.02% |
| EPA 20:5 n3 | 0.06% |
| DHA 22:6 n3 | 0.28% |
| Total n3 | 0.42% |
| Total n6 | 1.52% |
| Total Mono Unsaturated | 1.27% |
| Total Polyunsaturated Fats | 1.95% |
| Total Saturated Fats | 1.59% |

Calculated data uses information from typical raw material composition.  It could be expected that individual batches of diet will vary from this figure. **Diet post treatment by irradiation or auto clave could change these parameters**. We are happy to provide full calculated nutritional information for all of our products, however we would like to emphasise that these diets have been specifically designed for manufacture by Specialty Feeds.
