# Supplementary figures and images for: Long-term omega-3 supplementation modulates behavior, hippocampal fatty acid concentration, neuronal progenitor proliferation and central TNF-α expression in 7 month old unchallenged mice
Source: Front Cell Neurosci. 2014 Nov 21;8:399. doi: 10.3389/fncel.2014.00399 (PMC4240169; doi:10.3389/fncel.2014.00399)

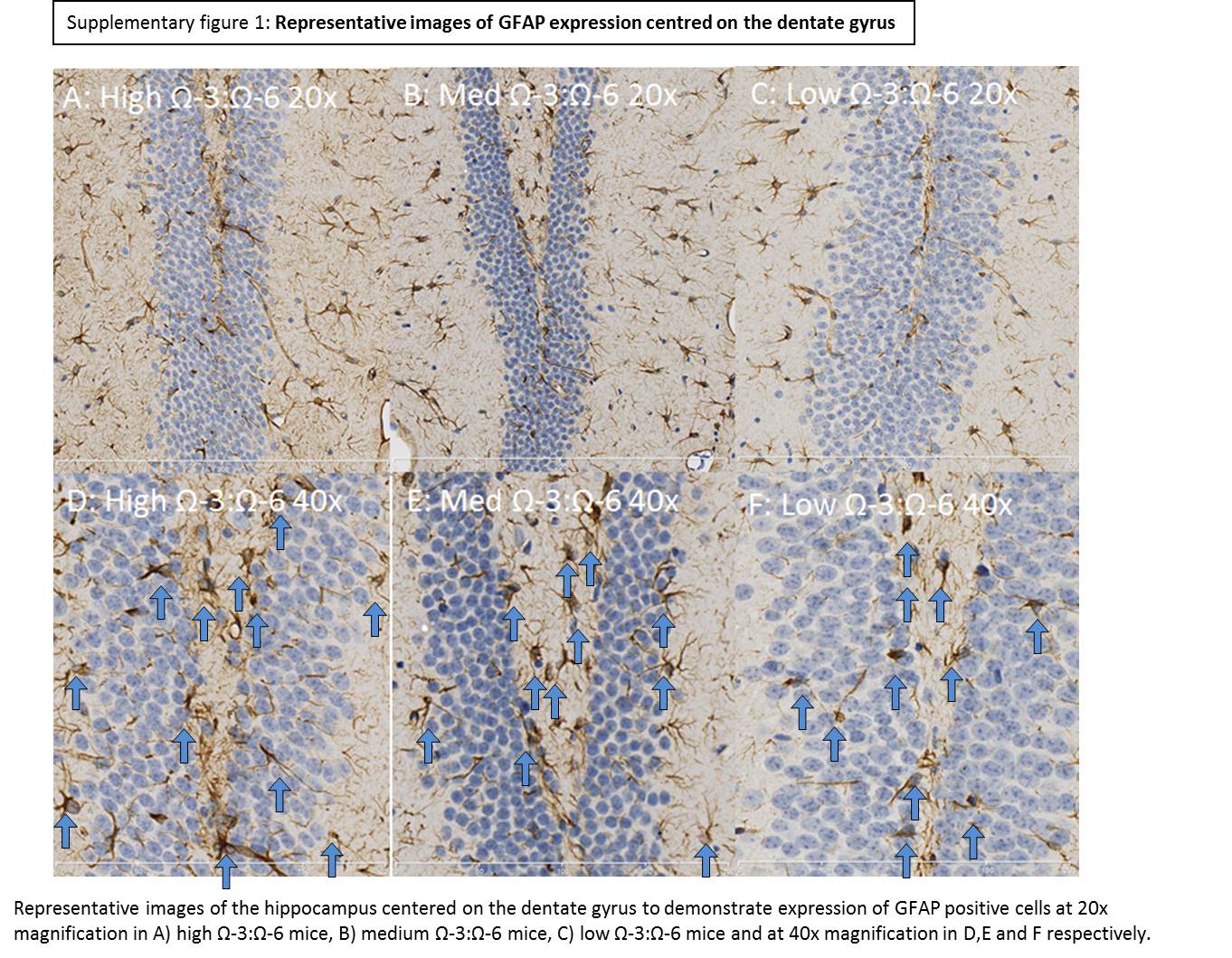

Supplement: Supplementary file 4 [file Image1.JPEG]

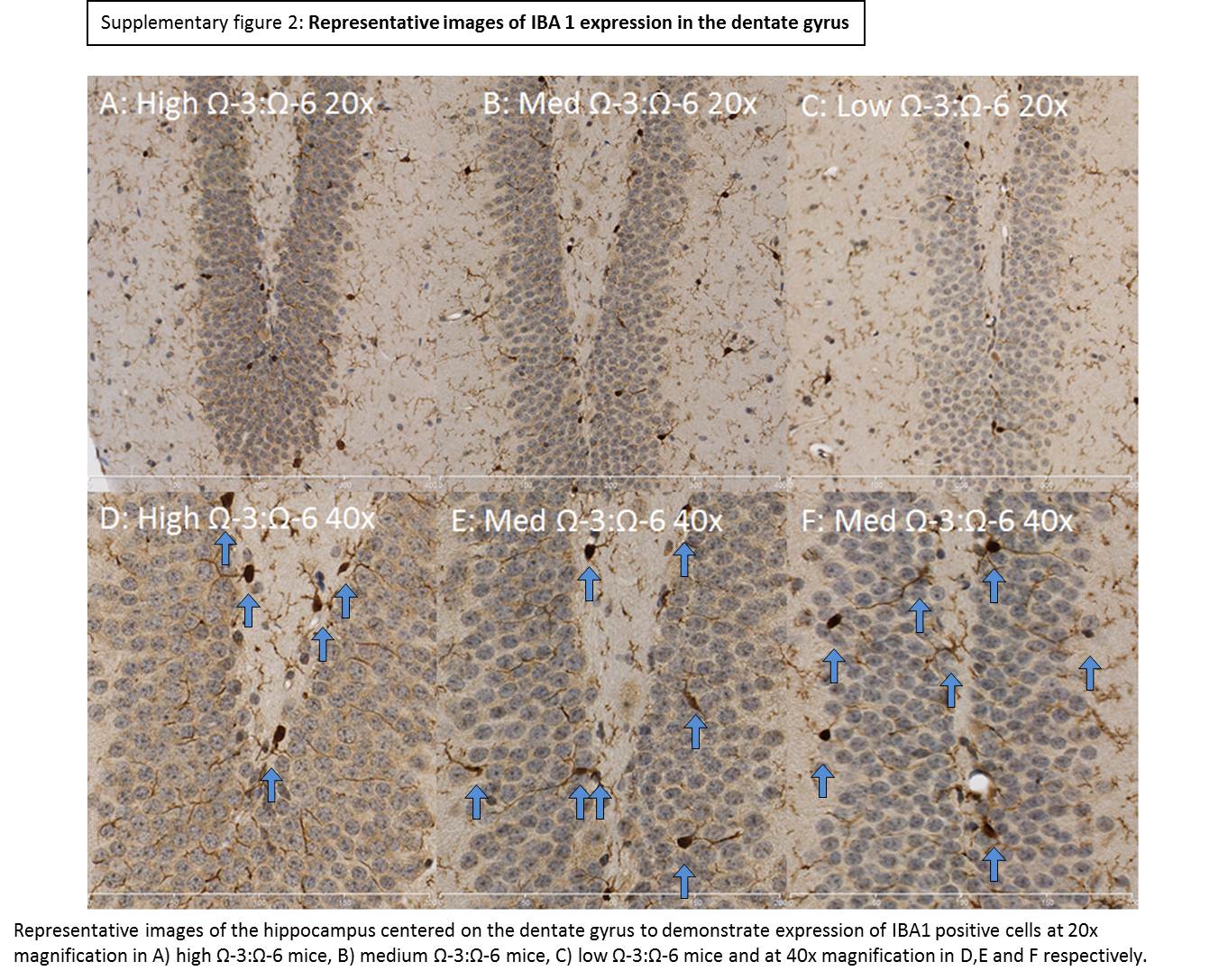

Supplement: Supplementary file 5 [file Image2.JPEG]

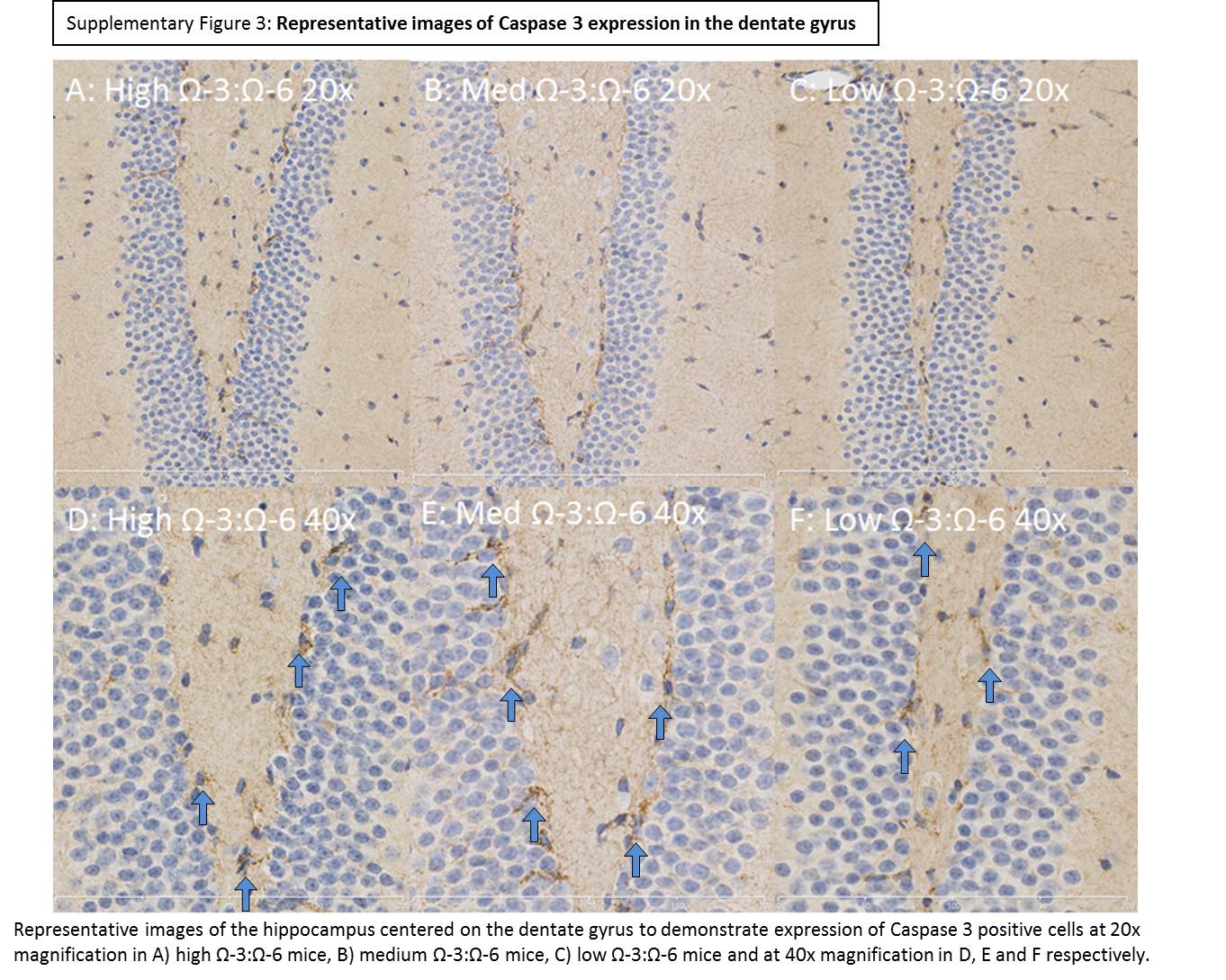

Supplement: Supplementary file 6 [file Image3.JPEG]
